# Radiofrequency catheter ablation of persistent atrial fibrillation in a patient with cor triatriatum sinister

**DOI:** 10.1016/j.hroo.2024.10.021

**Published:** 2024-11-06

**Authors:** Hideyuki Hasebe, Yoshitaka Furuyashiki, Kentaro Yoshida

**Affiliations:** 1 Department of Cardiology, Institute of Medicine, University of Tsukuba, Tsukuba, Japan; 2 Division of Arrhythmology, Shizuoka Saiseikai General Hospital, Shizuoka, Japan

**Keywords:** Ablation, Atrial fibrillation, Cor triatriatum sinister, Radiofrequency

**Key Findings**

▪ Cor triatriatum sinister (CTS) is a rare congenital condition often accompanied by atrial fibrillation (AF). Catheter ablation of AF in patients with CTS can be challenging due to the abnormal membrane.
▪ Catheter ablation of persistent AF using a radiofrequency catheter was successfully performed in a patient with CTS of Lucas classification C1a.
▪ Intracardiac echocardiographic images, computed tomographic images, and their merged images provide useful guidance for radiofrequency applications and the transseptal puncture procedure.

## Introduction

Cor triatriatum sinister (CTS) is a rare congenital condition in which a fibromuscular membrane divides the left atrium (LA) into 2 chambers. It is present in 0.1% to 0.4% of patients with congenital heart diseases.^1^ While the inferior and anterior chambers are connected to the LA appendage and mitral orifice, the superior and posterior chambers receive return blood flow from the pulmonary veins (PVs). In some cases, the abnormal membrane restricts blood flow from the superior and posterior chambers to the inferior and anterior chambers, leading to a mitral stenosis–like hemodynamic condition and PV congestion. Therefore, CTS is often accompanied by atrial fibrillation (AF).^2^ Catheter ablation in patients with CTS can be challenging due to the existence of the abnormal membrane and has not been well studied. We report a case of CTS of Lucas classification C1a in whom radiofrequency (RF) catheter ablation of AF was successfully performed.

## Case report

**Figure 1:**
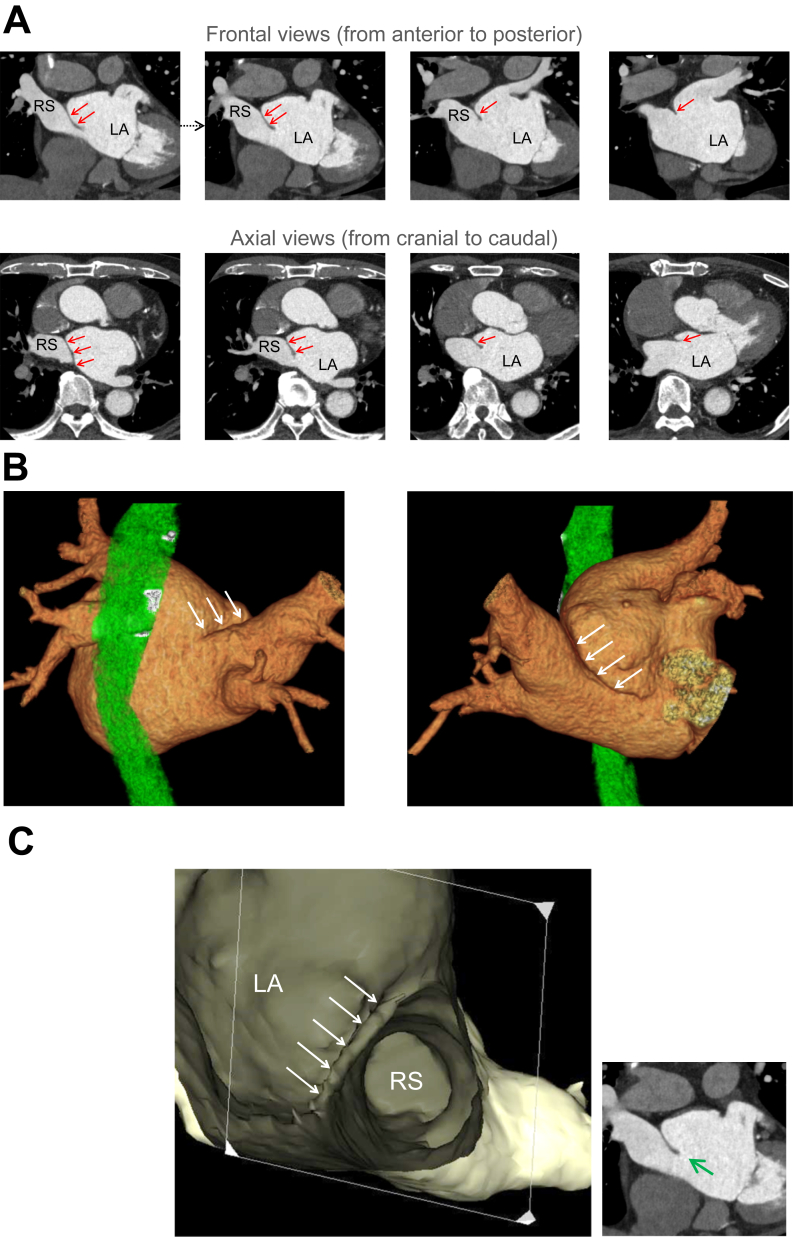
Frontal and axial computed tomographic views (A) and outer (B) and inner (C) 3-dimensional computed tomographic views showing an abnormal membrane (indicated by red or white arrows) dividing the left atrium (LA) into 2 chambers. In the inner view (C), the green arrow in the frontal view indicates the view direction. RS = right superior pulmonary vein.

A 65-year-old man with a history of hypertension and diabetes was referred to our institution for catheter ablation of symptomatic persistent AF for about 12 months. His symptoms were palpitations, and dyspnea on effort. Transthoracic echocardiography revealed no notable findings other than mild LA dilatation (47 mm). Three-dimensional multidetector computed tomography (MDCT) revealed that the LA was separated into 2 chambers by an abnormal membrane, and the right-sided PVs drained into the subdivision of the LA (Figure 1). These MDCT findings were consistent with CTS of Lucas classification C1a.

**Figure 2:**
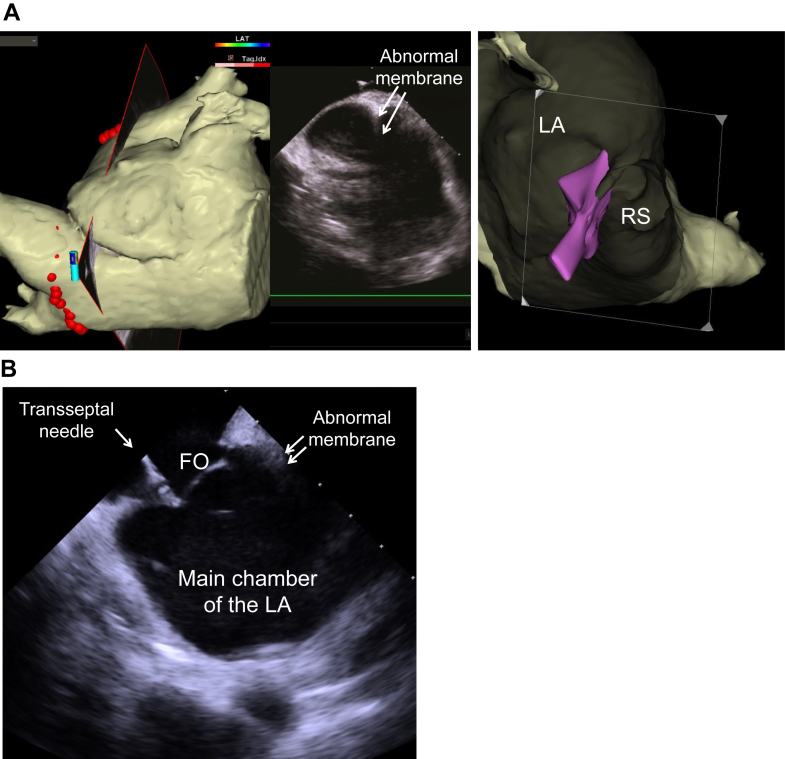
A: Intracardiac echocardiography shows the abnormal membrane subdividing the left atrium (LA) into 2 chambers and the CARTO merged image using the 3-dimensional computed tomography. B: Intracardiac echocardiography at the time of transseptal puncture. The septal-side edge of the membrane coincided with the upper edge of the fossa ovalis (FO). The entire FO faced the main chamber of the LA. RS = right superior pulmonary vein.

**Figure 3:**
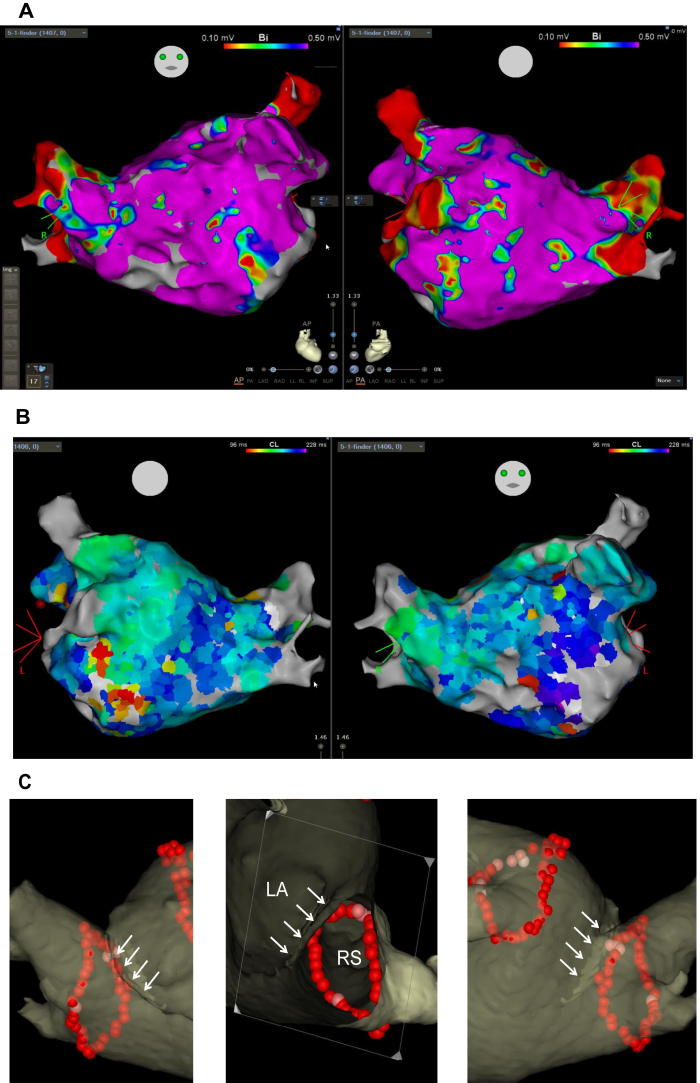
CARTO images. A: Voltage map in the left atrium (LA) during atrial fibrillation. B: CARTOFINDER atrial fibrillation cycle length map in the LA. C: The final lesion sets of extensive pulmonary vein isolation. RS = right superior pulmonary vein.

The ablation procedure was performed using a 3-dimensional navigation system (CARTO 3; Biosense Webster). We selected an RF catheter as the ablation tool considering the relatively large size of the right superior PV ostium (29 × 22 mm). Intracardiac echocardiography (SOUNDSTAR; Biosense Webster) detected the abnormal membrane, and the images were merged with the computed tomography images (Figure 2A). The septal-side edge of the membrane coincided with the upper edge of the fossa ovalis. The entire fossa ovalis faced the main chamber of the LA (Figure 2B). A transseptal puncture using an NRG RF Transseptal Needle (Baylis Medical) was easily performed without trouble. The mean pressures in the main chamber and subdivision of the LA were equivocal (20 and 21 mm Hg, respectively). After the transseptal puncture, the LA was mapped with CARTOFINDER using an Octaray catheter (Biosense Webster). During AF, no distinct low-voltage area (<0.5 mV) was observed on the voltage map in either the main chamber or subdivision of the LA (Figure 3A). The CARTOFINDER atrial fibrillation cycle length (AFCL) map revealed equivocal AFCLs in the main chamber and subdivision of the LA (Figure 3B). In addition, there were no apparent differences in the PV potentials between the right- and left-sided PVs.

Circumferential antral pulmonary vein isolation (PVI) was performed with RF applications in a point-by-point fashion using a ThermoCool SmartTouch irrigated-tip contact force–sensing ablation catheter (Biosense Webster). After the successful first-pass isolation of the left-sided PV, PVI for the right-sided PV was performed with an ablation index ≥520 in the anterior antrum and ≥450 in the other parts. Manipulations to pass the catheters beneath the membrane were often needed. Other than that, the procedure was performed as usual. First-pass isolation was also achieved in the right-sided PV (Figure 3C). In addition to PVI, RF energy was delivered at the focal activation sites detected in CARTOFINDER. All ablation procedures were successfully completed without complications. The patient has been free from atrial tachyarrhythmia and without any antiarrhythmic drugs for 6 months after the ablation session.

## Discussion

In CTS with a narrow fenestration, blood flow from the subdivision of the LA to the main LA can be restricted by the abnormal membrane.^2^^,^^3^ In our patient, such blood flow restriction seemed minimal because there was a large communication beneath the abnormal membrane on the MDCT images (Figure 1A). In fact, the mean pressures in the main chamber and subdivision of the LA were equivocal, indicating that the abnormal membrane had little impact on hemodynamics in this patient. Instead, hypertension, diabetes, and aging, common causes of AF, likely contributed the genesis of the AF. The AFCLs in the 2 chambers were also equivocal, suggesting that AF drivers equally existed in both chambers.

Although the abnormal membrane can be detected by transthoracic echocardiography in most patients with CTS, it could not be detected in parts of the patients like our patient. Enhanced MDCT may be considered prior to an ablation for AF in order to identify anatomic variants. Intracardiac echocardiography provided anatomical information regarding the abnormal membrane. Especially, the anatomical relationship between the abnormal membrane and fossa ovalis was important for transseptal puncture and the subsequent ablation procedures in the LA. If the abnormal membrane had extended below the upper margin of the fossa ovalis or the fossa ovalis faced the subdivision of the LA,^3^ catheter manipulation would have been more difficult.

Outcomes of AF ablation in patients with CTS were referred from previous case series and case reports.^2^^,^^4^^,^^5^ The AF-free rate after the index procedures in 14 previous cases was 87% with a mean follow-up period of 18.9 ± 26 months. Based on these data and the favorable outcome of our patient, the outcome of AF ablation in patients with CTS is likely equivalent to that in the usual patient with AF.

Considering the large size of the right superior PV in our patient, we chose RF ablation. During the procedure, catheter manipulation to access the right-sided PVs was not so difficult. If the PV size had been suitable, balloon-based PVI would have been feasible. However, creating a roof line would have been challenging due to the existence of the abnormal membrane regardless of the ablation tools used.

## Conclusion

Catheter ablation of persistent AF was successfully performed in a patient with CTS of Lucas classification C1a. Intracardiac echocardiographic images, MDCT images, and merging of these images served as useful guides for the RF applications and transseptal puncture procedure.

## Disclosures

The authors have no conflicts to disclose.
